# Correction: Nano copper(i) oxide/zinc oxide catalyzed *N*-arylation of nitrogen-containing heterocycles with aryl halides and arylboronic acids in air

**DOI:** 10.1039/d2ra90095e

**Published:** 2022-09-21

**Authors:** Mona Hosseini-Sarvari, Fatemeh Moeini

**Affiliations:** Department of Chemistry, Shiraz University Shiraz 71454 I. R. Iran hossaini@shirazu.ac.ir +98 711 6460788 +98 711 6137169

## Abstract

Correction for ‘Nano copper(i) oxide/zinc oxide catalyzed *N*-arylation of nitrogen-containing heterocycles with aryl halides and arylboronic acids in air’ by Mona Hosseini-Sarvari *et al.*, *RSC Adv.*, 2014, **4**, 7321–7329, https://doi.org/10.1039/C3RA46548A.

The authors regret considerable unattributed text, figure and data overlap between their article in *RSC Advances* and ref. 1. Due to the overlap in the synthesis and characterisation of the Cu_2_O/ZnO nanoflake, ref. 1 should have been cited in this paper.

Both articles contain some unique data, however, Fig. 1–4 and Table 1 of the *RSC Advances* article were re-used from ref. 1 without being correctly attributed. Tables 2–7 in this *RSC Advances* article were not published elsewhere.

The corrected captions are shown below:

Fig. 1 FT-IR spectrum of Cu_2_O/ZnO nanoflake. Reproduced from ref. 1.

Fig. 2 The XRD pattern of Cu_2_O/ZnO nanoflake. Reproduced from ref. 1.

Fig. 3 The SEM image of Cu_2_O/ZnO nanoflake. Reproduced from ref. 1.

Fig. 4 TEM image of Cu_2_O/ZnO nanoflake. Reproduced from ref. 1.

Table 1 Results of BET surface area measurements for Cu_2_O/ZnO nanoflake. Reproduced from ref. 1

The Royal Society of Chemistry apologises for these errors and any consequent inconvenience to authors and readers.

## Supplementary Material
